# Posterior reversible encephalopathy syndrome with ischemic infarction complicated with intrauterine fetal death: A case report

**DOI:** 10.1097/MD.0000000000018877

**Published:** 2020-01-24

**Authors:** Wei-Chih Yeh, Li-Min Liou, Meng-Ni Wu

**Affiliations:** Department of Neurology, Kaohsiung Medical University Hospital, Kaohsiung, Taiwan.

**Keywords:** posterior reversible encephalopathy syndrome, seizure, encephalomalacia

## Abstract

**Rationale:**

Posterior reversible encephalopathy syndrome (PRES), a rare neurologic disorder, manifests as headache, altered mental status, seizures, visual disturbances, and other focal neurologic signs with typically reversible clinical symptoms and image changes. Although the underlying mechanism remains unknown, a current theory indicates cerebral autoregulation failure as the primary cause. We report a case of PRES with stroke in an adult with intrauterine fetal death (IUFD).

**Patient concerns:**

A 35-year-old Asian woman with twin pregnancy underwent cesarean section at 32 weeks of gestation because of IUFD. She presented with focal seizures and visual field defect 2 days after undergoing cesarean section. Her blood pressure and kidney, liver, and coagulation functions were normal without proteinuria.

**Diagnosis:**

PRES was diagnosed based on a series of brain magnetic resonance imaging findings. Ischemic infarction in the right frontal lobe eventually developed to encephalomalacia.

**Interventions:**

The patient received levetiracetam and valproate for seizure management.

**Outcomes:**

Five days after the onset, seizures were under control. All neurologic deficits completely improved after 21 days of admission.

**Lessons:**

PRES can occur in women with IUFD without preeclampsia or eclampsia symptoms. Although most cases result in vasogenic edema of the brain and exhibit good prognosis, PRES can cause cytotoxic edema and permanently damage the brain.

## 1 Introduction

Posterior reversible encephalopathy syndrome (PRES) was first described in 1996 by Hinchey et al.^[1]^ The clinical diagnosis of PRES comprises headache, seizures, encephalopathy, and visual disturbances.^[1,2]^ PRES is characterized by neuroimaging findings of reversible vasogenic subcortical edema without infarction.^[2]^ Although the mechanism underlying PRES remains unclear, the most widely accepted hypothesis is that severe hypertension causes blood–brain barrier breakdown, which eventually causes impaired cerebral autoregulation.^[2]^ Typically, PRES is most commonly associated with hypertensive encephalopathy, preeclampsia or eclampsia, renal disease, sepsis, and chemotherapy exposure.^[2,3]^ Usually, PRES causes reversible vasogenic edema in the brain but rarely causes ischemic infarctions and eventually encephalomalacia.^[3]^

To the best of our knowledge, there are no studies reporting PRES in a patient with intrauterine fetal death (IUFD) without preeclampsia or eclampsia. Here, we report a case of a patient diagnosed with PRES complicated by IUFD without preeclampsia or eclampsia and permanent encephalomalacia after the improvement of clinical symptoms.

## 2 Presenting concerns

### 2.1 Patient information

A 35-year-old woman with twin pregnancy (2nd gravidity, with 1 spontaneous abortion) was admitted to a local hospital for tocolysis because of suspected preterm labor at 32 weeks of gestation. She was healthy and reported no diseases in the past. After 5 days of admission, she underwent an emergent cesarean section for IUFD following the loss of heartbeat in 1 fetus.

### 2.2 Clinical findings

Two days after the cesarean section, she experienced a sudden onset of consciousness disturbance, which was followed by generalized limb convulsions, lasting for a few minutes. She was transferred to the emergency department of our hospital for further management.

Limb convulsions stopped after the intravenous administration of 1-mg lorazepam and 1000-mg levetiracetam. The patient exhibited drowsiness with bilateral gaze limitation to the right, binocular left visual field defect, and left-side hemiparesis. In addition, intermittent left-side limbs twitching persisted despite the intravenous administration of 1000-mg levetiracetam (twice/d). Status epilepticus was suspected; levetiracetam was titrated to 1500 mg (twice/d), and she was admitted to the intensive care unit on the day of seizure onset. Furthermore, oral aspirin (100 mg/d) was prescribed for a possible ischemic stroke with 60 mL mannitol every 6 hours. Her blood pressure upon admission was normal (107/75 mm Hg). Moreover, urinalysis revealed the absence of proteinuria.

Five days after seizure onset, electroencephalography revealed diffuse background slowing at 5 to 7 Hz with runs of lateralized periodic diphasic and triphasic epileptiform discharges in the right hemisphere. Accordingly, 400-mg valproate (thrice/d) was included in the treatment regimen. Subsequently, no recurrence of seizure was noted; however, drowsiness with incoherent speech and left hemiparesis persisted. Laboratory data revealed the following: leukocytosis (11,830/μL), elevated C-reactive protein level (27.73 mg/L), and elevated creatine phosphate kinase level (347 IU/L); however, renal and liver functions were within normal limits. In addition, autoimmune profiles, including antinuclear antibody, rheumatic arthritis factor, complement 3 and 4 levels, and antiphospholipid survey were within normal limits. Furthermore, coagulopathy profiles, including homocysteine, serum viscosity, fibrinogen, antithrombin III, protein C, and protein S levels, were unremarkable. Holter electrocardiography revealed sinus rhythm, and transthoracic cardioechography revealed neither remarkable vegetation nor valvular heart disease. Furthermore, carotid duplex revealed normal blood flow over the bilateral carotid and vertebrobasilar system.

### 2.3 Diagnostic focus and assessment

**Figure 1:**
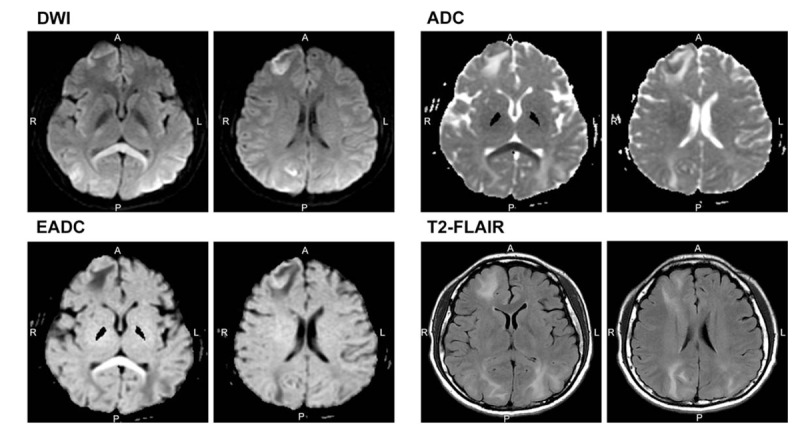
Brain magnetic resonance imaging (MRI) shows high-signal intensity lesions on diffusion-weighted imaging (DWI), exponential apparent diffusion coefficient, and T2 fluid-attenuated inversion recovery (T2-FLAIR) images in the right frontoparietal, temporal, occipital lobes and left temporal, parietal, and occipital lobes, splenium of the corpus callosum with gyral enhancement. Apparent diffusion coefficient (ADC) series also show high-signal intensity in the aforementioned cortex area. However, low-signal intensity is observed in the right frontal area and the splenium of the corpus callosum, indicating cytotoxic edema at these 2 areas.

Brain computed tomography scan conducted on the day of seizure revealed a hypodense lesion in the right frontoparietal and occipital lobes. On day 9 after the onset of seizure, brain magnetic resonance imaging (MRI) revealed high-signal intensity lesions on diffusion-weighted imaging (DWI), T2-weighted imaging, and T2 fluid-attenuated inversion recovery (FLAIR) in the right frontal, temporal, occipital, and parietal lobes; left temporal, occipital, and parietal lobes; and splenium of the corpus callosum, respectively, with gyral enhancement but without any evidence of cerebral venous sinus thrombosis (Fig. 1).

**Figure 2:**
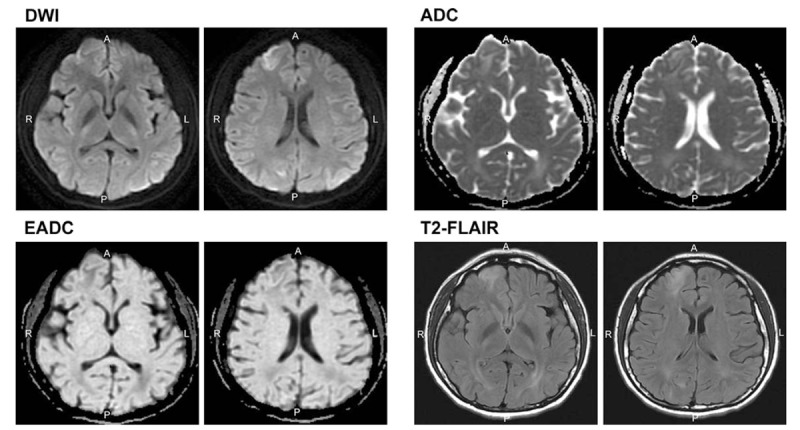
Persistent high-signal intensity lesions on diffusion-weighted imaging (DWI), T2 fluid-attenuated inversion recovery (FLAIR) in the right frontal and parietal lobes. Decreased signal intensities are observed on apparent diffusion coefficient (ADC) maps in the right temporo-occipital and splenium of the corpus callosum, suggesting a partial reversal of vasogenic edema. Total reversal of cytotoxic edema is observed in the splenium of the corpus callosum.

**Figure 3:**
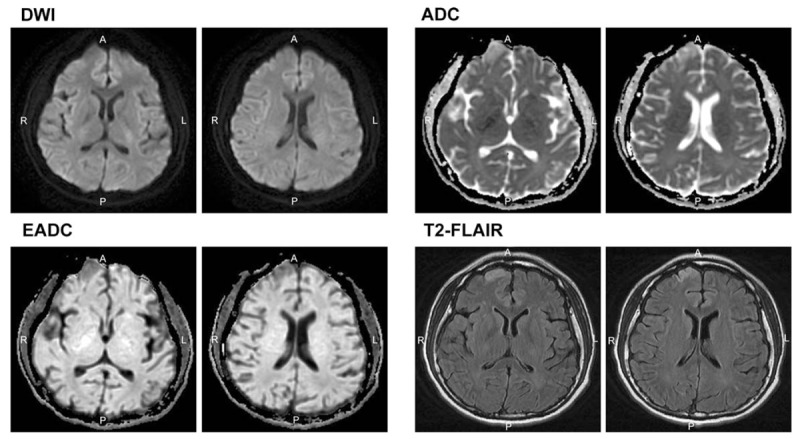
Brain magnetic resonance imaging (MRI) 2 months later showed high signal on T2-weighted (T2W) and fluid-attenuated inversion recovery (T2-FLAIR) images and low-signal intensity on T1-weighted (T1W) images in the right frontal lobe, suggesting encephalomalacia.

Twenty-one days after the onset of seizure, brain MRI revealed persistent high-signal intensity lesions on DWI and T2-FLAIR in the right frontal and parietal lobes (Fig. 2). Two months after the onset of seizure, all neurologic deficits completely improved. Follow-up MRI revealed the resolution of all signal changes, except for one hypersignal in the right frontal lobe (Fig. 3).

The patient was diagnosed with PRES based on the clinical presentation of acute-onset seizures, encephalopathy, and visual disturbances, accompanied by reversible vasogenic subcortical edema according to a series of brain MRI.

### 2.4 Follow-up and outcomes

Five days after the onset, seizures were under control. All neurologic deficits completely improved after 21 days of admission. The patient was discharged after 25 days of admission and remained asymptomatic for 1 year after discharge from the hospital.

## 3 Discussion

Reportedly, PRES can be triggered by eclampsia, hypertensive emergency, or exposure to immunosuppression.^[2,3]^ The mechanism of PRES is not completely understood, and 2 hypotheses are proposed. First, severe hypertension greater than the upper limit of cerebral vascular autoregulation leads to hyperperfusion and vasogenic edema. Second, endothelial dysfunction caused by circulating endogenous or exogenous toxins leads to vasoconstriction and hypoperfusion and consequently ischemic edema.^[4]^

Fetal death is defined as death before the complete expulsion or extraction from the mother of a product of human conception, irrespective of the duration of pregnancy that is not induced by the termination of pregnancy.^[5]^ A previous case report has shown the occurrence of neuromyelitis optica during pregnancy complicated by PRES, eclampsia, and fetal death.^[6]^ Our patient presented with neither hypertension nor proteinuria, and no coagulopathy was observed. Moreover, her autoimmune profiles including phospholipid antibody levels and complement system were normal. Another case report found PRES in a pregnant patient without preeclampsia,^[7]^ although they did not mention the underlying diseases, and no further survey of autoimmune profile and coagulopathy was conducted in the study.

Studies on animal models have shown that a decrease in uterine perfusion pressure can cause PRES.^[8]^ However, to date, no study has shown the direct association between IUFD and PRES in a woman without any prepartum complications.

The spectrum of imaging findings in PRES is extensive, and the involvement of the frontal lobe, temporal lobe, and cerebellar hemispheres is common, along with the occasional presence of lesions in the brainstem, basal ganglia, deep white matter, and splenium.^[9,10]^

Fugate et al have presented the following criteria for the diagnosis of PRES: neurologic symptoms of acute onset, neuroimaging abnormalities, including focal vasogenic edema, and reversible clinical and/or radiologic findings.^[10,11]^

Typically, PRES is reversible; however, lesions can progress to irreversible damage and encephalomalacia.^[2,12]^ In the present case, the patient exhibited vasogenic and cytotoxic edemas in different brain areas. Vasogenic edema improved after proper management, as in most reported cases.^[12,13]^ However, the cytotoxic lesion of the splenium of the corpus callosum can be entirely reversed without neurologic sequelae, and the right frontal lobe cytotoxic lesion eventually results in tissue loss. A study has reported that apparent diffusion coefficient (ADC) maps and DWI can successfully differentiate PRES from early cerebral ischemia, thereby playing a vital role in making treatment decisions.^[12]^ In addition, high-signal intensity on DWI and low ADC values are correlated with cerebral infarction and may represent the earliest signs of nonreversibility because severe vasogenic edema progresses to cytotoxic edema.^[12,13]^

Some case reports have described postpartum PRES. For example, Zhang et al^[14]^ have reported a case of PRES secondary to delayed maternal postpartum eclampsia. Lio et al^[15]^ showed a case report of PRES complicated by postpartum hemorrhage. Shi et al^[16]^ presented a case of postpartum hemorrhage after a normal delivery requiring uterine artery embolization and the development of PRES 2 hours after the procedure. All these case reports have indicated that severe postpartum complications preceded PRES; however, in the present case, the patient presented with neither preeclampsia nor eclampsia and elevated blood pressure and proteinuria were not observed during hospitalization.

The occurrence of PRES complicated with ischemic stroke is rare. Liang et al^[17]^ reported a case of an isolated pons variant of PRES complicated with ischemic stroke in a young patient. Imataki et al^[18]^ reported a case of reversible cerebral vasoconstriction syndrome followed by PRES, which progressed to cerebral infarction because of tacrolimus administration. To the best of our knowledge, this is the first case of PRES with stroke in a patient with IUFD.

In conclusion, this report reveals that PRES can occur after delivery without the symptoms of preeclampsia or eclampsia and cause permanent encephalomalacia. Therefore, PRES must be considered as a differential diagnosis in pregnant women with IUFD presenting neurologic deficits.

## Acknowledgment

The authors thank Enago (www.enago.com) for the English language review.

## Author contributions

**Writing – original draft:** Wei-Chih Yeh, Li-Min Liou, Meng-Ni Wu.

**Writing – review & editing:** Wei-Chih Yeh, Li-Min Liou, Meng-Ni Wu.
